# Neural mechanisms linking Western diet consumption and cognitive impairment: a role for the vagus nerve

**DOI:** 10.1042/CS20250034

**Published:** 2026-05-28

**Authors:** Anna M.R. Hayes, Alexander G. Bashaw, Molly E. Klug, Scott E. Kanoski

**Affiliations:** 1 Department of Biological Sciences, Human and Evolutionary Biology Section, University of Southern California, Los Angeles, CA 90089, U.S.A.; 2 Department of Food Science and Technology, Oregon State University, Corvallis, OR 97331, U.S.A.; 3 Neuroscience Graduate Program, University of Southern California, Los Angeles, CA 90089, U.S.A.

**Keywords:** acetylcholine, dopamine, high fat diet, neuromodulators, sugar, vagus nerve

## Abstract

Consumption of a Western diet containing large proportions of calories from highly palatable foods that are high in fat and sugar is causally linked with obesity and metabolic dysfunction. In addition to these widely known deficits, Western diet consumption also negatively impacts various cognitive domains, including learning and memory function (e.g. Alzheimer’s and related dementias), affective processes (e.g. anxiety, depression), and reward-related behaviors. However, the underlying neurobiological mechanisms that link Western diets to impaired cognition are poorly understood. In this review, we highlight recent findings revealing the impacts of Western diet consumption on the molecular machinery and signaling of key central neuromodulators (acetylcholine, dopamine, serotonin) that contribute to the dysregulation of fundamental cognitive and behavioral processes. Based on emerging evidence, we propose that Western diet-associated perturbations in central neuromodulator signaling and associated neurocognitive impairments are driven, at least in part, by blunted gut-to-brain communication through the vagus nerve. While future research is necessary to fully elucidate this hypothesized connection, it provides a framework to advance our understanding of the mechanisms mediating diet-cognition interactions.

## Introduction

Consumption of a ‘Western diet’ (WD) containing hyperpalatable and processed foods that are high in saturated fats and refined carbohydrates is strongly linked with obesity [1–4]. Over 40% of individuals in the United States are now classified as obese [5] and the rates of obesity are rising globally [6]. Of particular concern is the rising prevalence of obesity in children and adolescents, tripling from 2.0% in 1990 to 6.8% in 2021 [7], which predisposes these individuals to a host of health consequences throughout the lifespan [8,9]. In addition to its association with negative metabolic and cardiovascular outcomes, obesity in midlife is a risk factor for mild cognitive impairment and dementia, including Alzheimer’s disease and vascular dementia [10–12]. The number of people living with dementia worldwide is projected to increase from nearly 60 million today to over 150 million in 2050, in part due to the high percentage of aging adults currently classified as overweight or obese [13,14]. Developing novel approaches to prevent and treat both obesity and dementia, as well as to understand the role of neurodevelopmental antecedents, is critical to reduce the health and economic burdens associated with these comorbid conditions.

Although our understanding of the etiologies of obesity and dementia is incomplete, both have been strongly associated with consumption of a WD [1–4,8,15–17]. In addition to dementia, both WD consumption and obesity are linked with affective disorders and disruptions in behaviors relating to reward and motivation in mammalian species, including both humans and experimental rodent models [18–23]. Furthermore, increasing evidence indicates that WD consumption during early life periods negatively impacts neurocognitive development [24–28]. In this review, we discuss the current state of evidence on the neural mechanisms implicated in WD-induced impacts on cognition, focusing on the cognitive domains of memory, anxiety-like behavior, depressive-like behavior, and reward-associated behaviors. We propose that consumption of a WD, particularly during adolescence, yields deleterious effects on brain-derived signaling profiles of critical neuromodulators (e.g. acetylcholine (ACh), dopamine (DA), serotonin (5-HT)) that regulate fundamental cognitive processes. Further, we emphasize that these alterations in neural systems may be, at least in part, secondary to WD-associated impairments in vagus nerve to brain signaling.

## Vagus nerve: a conduit of Western diet-induced cognitive deficits

The vagus nerve facilitates bidirectional communication between peripheral organs (e.g. the gastrointestinal tract) and the brain, and thus is considered a key conduit of the gut–brain axis [29]. WD consumption modulates direct vagal structural innervation of the caudal brainstem, which is where the vagus nerve first connects to the brain [30–32]. For example, in rats, 7-day consumption of a high-fat diet (HFD; 60% kcal from fat) decreases density of vagal afferent terminals in the nucleus of the solitary tract (NTS) compared to a low-fat diet (LFD; 19% kcal from fat) [30]. Contrarily, the same study also showed that 21 days of HFD consumption increases vagal afferent terminal density in the NTS (versus LFD consumption), signifying that a HFD can lead to time-dependent and dynamic vagal remodeling of brainstem inputs [30]. In addition to diets high in fat, consuming refined carbohydrates can also affect vagal afferent organization, as 28-day consumption of a low-fat, high-sucrose diet (10% kcal from fat, 70% kcal from carbohydrate) or a high-fat, high-sucrose diet (45% kcal from fat, 35% kcal from carbohydrate, predominantly sucrose) led to decreased vagal afferent terminal density in the NTS compared to a control low-fat, low-sucrose diet (13.5% kcal from fat, 58% kcal from carbohydrate, predominantly starch) in rats [31]. Although further characterization of the extent of these structural changes is warranted, these studies suggest that diets high in fat and/or sugar—key characteristics of a WD—can reshape the structure of vagus nerve input from the gut to the brain.

The WD-induced blunting of vagal afferent signaling contributes to impaired satiation and satiety processes, ultimately contributing to overeating [29]. This is mediated in part by decreased sensitivity to food intake-reducing capacity of gut-derived satiation peptides released during a meal that signal via the vagus nerve to the brain, like cholecystokinin (CCK). For example, HFD (34% kcal from fat) consumption in rats attenuates the capacity of peripheral CCK administration to engage immediate early gene expression (c-Fos marker of neural activation) in the NTS (where vagal afferents synapse), results accompanied by a weaker reduction in food intake typically caused by CCK injections in HFD-fed rats relative to LFD-fed controls [33]. Similarly, vagal afferent neuron (VAN, a.k.a., nodose ganglia) sensitivity to CCK, 5-hydroxytryptamine (5-HT, or serotonin), and intestinal distension, measured via VAN electrophysiological responses, is attenuated in rats fed a 60% kcal HFD relative to a low-fat (10% kcal from fat) control diet [34]. On the other side of the energy balance equation, HFD consumption also impacts VAN responses to orexigenic gut signals, as a HFD (60% kcal from fat) augments the inhibitory effect of ghrelin, an appetite-promoting stomach-derived hormone, on distension-induced effects on VAN tension receptors [32]. Signaling by the adipocyte-derived anorexigenic hormone leptin is also blunted by diet-induced obesity, which may precede and be causally related to the effects of HFD on reduced VAN sensitivity to CCK [35]. While the mechanisms mediating WD-associated changes in vagal structure and signaling are incompletely understood, it is possible that these outcomes are reflective of compensatory responses to nutrient-driven overstimulation through HFDs or diets that are both high in fat and sugar [36].

**Figure 1:**
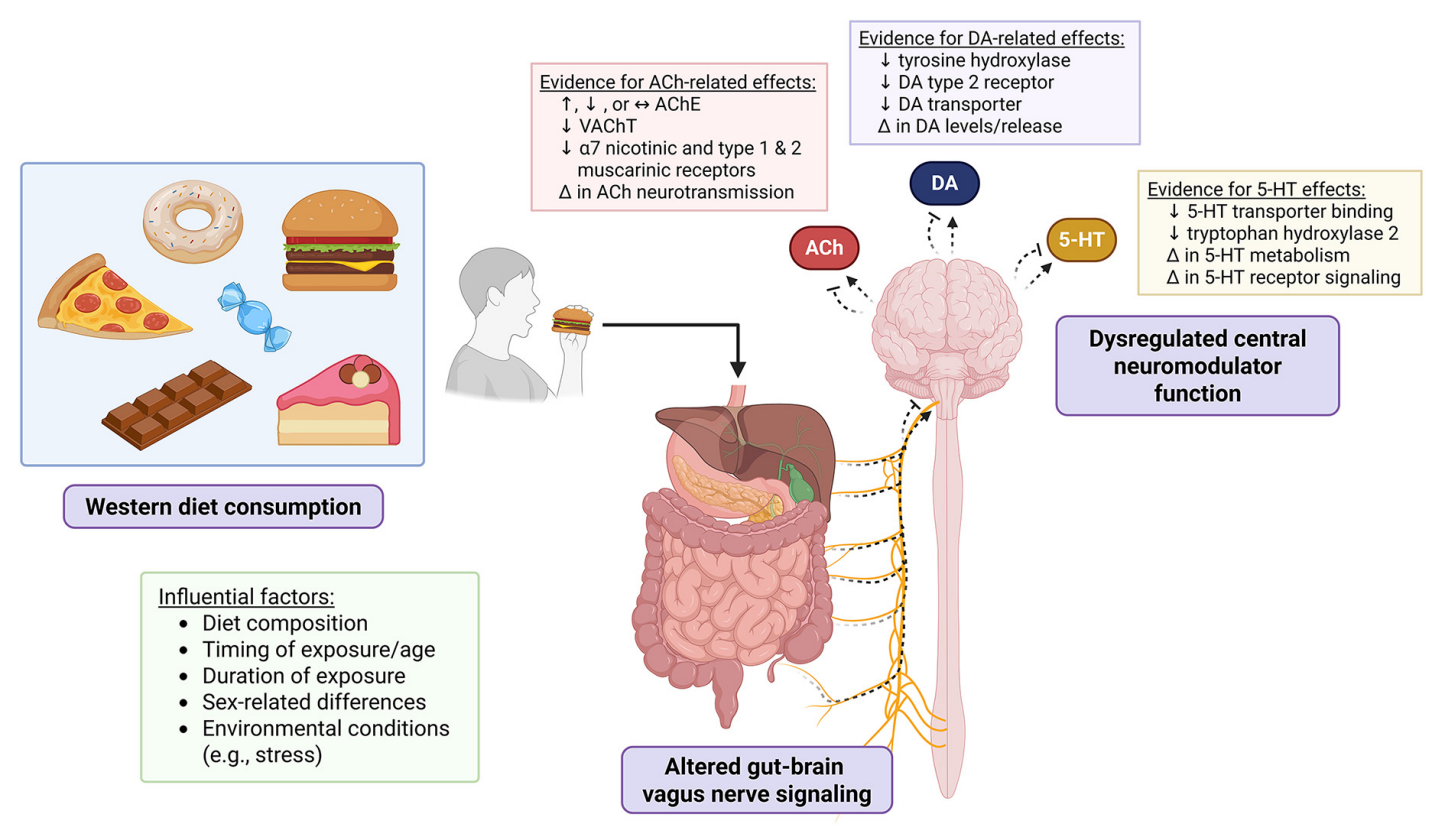
Schematic conceptual diagram for Western diet-induced impairments in cognitive function We propose that consumption of a WD imparts changes in vagus gut-to-brain signaling that ultimately modulate acetylcholine, DA, and serotonin function in the brain, resulting in altered learning and memory function, motivated behavior, anxiety-like behavior, and/or depression-like behavior. The illustrated acetylcholine-related effects have largely been observed in the hippocampus; DA-related effects in the striatum, locus coeruleus (LC), and nucleus accumbens; and serotonin-related effects in the hypothalamus and dorsal raphe. Dotted curves with arrows and inhibitor lines indicate the varied responses observed (increase, decrease, no change in signaling), and deltas (∆) represent alterations in observed outcomes. ACh, acetylcholine; AChE, acetylcholinesterase; DA, dopamine; 5-HT, serotonin; VAChT, vesicular acetylcholine transporter; WD, Western diet. Created in https://BioRender.com.

The overarching hypothesis of this review is that consuming a WD (or components of a WD) negatively impacts cognitive function, in part, by altering vagus nerve signaling (Figure 1). While none of the studies described above examined the effects of dietary exposures on cognitive outcomes, a growing body of evidence indicates that vagus nerve function can influence multiple modalities of cognition, such as memory, depressive-like behavior, and motivated behavior [37–42]. While this functional connection between WD, impaired vagal signaling, and neurocognitive outcomes remains speculative, Chunchai and colleagues [38] directly explored the effects of vagus nerve stimulation (VNS) on cognitive function in HFD (59% kcal from fat)-induced obese rats. VNS treatment was facilitated via a bipolar cuff electrode implanted around the left cervical vagus nerve to deliver repeated cycles of stimulation (14 s stimulation, 48 s rest) for 12 weeks versus the sham group that received the same devices but no stimulation. At baseline, HFD consumption impaired spatial learning and memory as assessed by the Morris Water Maze test (versus consumption of a normal diet containing 20% kcal from fat). Twelve weeks later, VNS prevented any further cognitive decline due to the HFD, indicating that chronic stimulation of the vagus nerve can offset diet-induced memory impairments. Although these effects were not accompanied by differences in body weight between sham and VNS-treated rats, VNS treatment lowered visceral fat levels and improved glucose tolerance and plasma lipid profiles, suggesting a potential complex interplay between obesity-related comorbidities, the vagus nerve, and memory function. Complementing these results, throughout the present review we highlight emerging neural mechanisms linking WD consumption to impairments in various cognitive domains (particularly via altered neurotransmitter systems), while interpreting these findings within a framework purporting that vagal-brain signaling is a primary functional anchor mediating diet-cognition interactions.

## Acetylcholine

A growing body of evidence indicates that dietary factors can alter acetylcholine (ACh) function in the brain, which can affect various behavioral outcomes. A population of ACh-producing neurons resides in the medial septum [43,44], which functions as an anatomical intermediary between the brainstem (where vagal afferents synapse) and the dorsal subregion of the hippocampus (HPC) [39]. ACh plays a critical role in learning and memory, including dementia pathology [45,46]. For example, dietary supplementation of choline, a precursor of ACh, reduces memory impairments in mouse models of Alzheimer’s disease [47,48], and consumption of a WD impairs ACh signaling and HPC-dependent memory function in mice and rats [49,50]. We recently revealed that *ad libitum* consumption of a cafeteria-style WD during early life (postnatal days 26–56; diet consisting of a rodent chow containing 45% kcal from fat plus an assortment of high-fat, high-sugar (HFHS) foods; overall consumption of 44% kcal from fat) versus standard rodent chow (13.5% kcal from fat) in rats resulted in long-lasting memory impairments in the hippocampal-dependent novel object in context test, and that such memory impairments persisted despite a healthy diet intervention during early adulthood [49]. Importantly, these memory deficits were driven by dysregulated ACh neurotransmission in the HPC, as revealed by *in vivo* fiber photometry measures showing blunted ACh release during a memory challenge, and further, by reduced vesicular ACh transporter (VAChT) levels in the dorsal HPC. Subsequent pharmacology experiments revealed that α7 nicotinic ACh receptor (α7nAChR) is a key intermediary of the WD effects on hippocampal dysfunction, as α7nAChR agonism reversed WD-associated memory impairments [49]. While ACh-based treatments for Alzheimer’s disease have been pursued for many years with limited success [51,52], these preclinical findings suggest that there could be important differences in neurodevelopment-driven cognitive impairments and later life Alzheimer’s disease pathology, and/or developmental/timing-specific windows for ACh-based strategies targeting treatment versus prevention.

Consistent with the findings from our lab, a recent study by Ramírez-Cruz and colleagues [50] in adult rats with 12-week consumption of a HFD (42% kcal from fat) and a high-sugar beverage (40% sucrose w/v) found that the WD group exhibited impaired spatial learning and memory in the Morris Water Maze test. For this diet model, the WD increased adiposity, disrupted blood-brain-barrier (BBB) permeability, and increased the activity of acetylcholinesterase (AChE; the enzyme that synaptically degrades ACh) in the HPC. The authors hypothesized that the WD promoted overactivity of AChE, which in turn led to increased/faster hydrolysis of ACh upon release at cholinergic synapses, ultimately yielding impaired memory function. Ramírez-Cruz and colleagues [50] also found that administration of nicotinamide (an uncompetitive inhibitor of AChE) in drinking water (5, 10, and 15 mM) during the 12-week diet period prevented the negative WD-induced effects on adiposity, BBB integrity, memory, and AChE activity.

Although these and other recent findings clearly implicate altered ACh signaling in WD-induced memory deficits, the effects of WD on specific ACh signaling mechanisms vary across studies. Kosari and colleagues [53] found that 12-week consumption of a very HFD (81% kcal from fat) or WD (41% kcal from fat) impaired spatial memory as assessed via the Y-maze task, yet neither diet altered AChE levels in the HPC and only the very HFD resulted in increased AChE signaling in the striatum. In contrast, Kaizer and colleagues [54] found decreased AChE activity in the HPC, hypothalamus, and cerebral cortex in rats after ∼26-week consumption of high-sugar (68% kcal from carbohydrate, 21% kcal from refined sugar and 47% kcal from starch) or high-fat (75% kcal from fat) diets in adult rats. Similarly, adult rats receiving a HFD (45% kcal from fat) versus chow (7% kcal from fat) for 17 weeks had lower levels of AChE in both the HPC and frontal cortex [55]. Differences in the type of diets used (e.g. variations in % calories from fat and sugar), diet duration, age during diet exposure, and even type of quantification of AChE (e.g. protein versus gene expression) may account for some of these discrepancies. Collectively, however, it appears that WD-associated memory impairments are accompanied by alterations in ACh signaling, generally in the direction of reductions in ACh signaling.

ACh receptors include both nicotinic and muscarinic subtypes, and WD consumption has been shown to affect the expression of both subtypes within the brain. We found that α7nAChR agonism rescued WD-induced memory impairments in an early life WD model [49], thus highlighting a functional role for nicotinic ACh receptors in mediating WD-associated hippocampal dysfunction. Similarly, Martinelli and colleagues [55] found reduced protein levels of α7nAChR in the HPC after 17 weeks of HFD consumption, further highlighting that this receptor subtype is particularly susceptible to dietary insults. Furthermore, the expression of both types 1 and 3 muscarinic receptors was reduced in the HPC of adult rats that received ∼13-week exposure to an HFD (56% kcal from fat) versus standard chow (10% kcal from fat) [56]. Overall, an accumulating body of evidence supports a critical role of both nicotinic and muscarinic ACh receptors in WD-induced memory impairments. Additional research is needed to clarify the influence of nicotinic versus muscarinic receptors (separately) versus a combination of these receptors in mediating such deficits, which may guide future pharmacotherapy for memory disorders.

### Vagus nerve hypothesis related to acetylcholine

Yet unknown is precisely how WD consumption leads to impaired ACh neurotransmission in the HPC. Our group revealed that ablation of gastrointestinal-specific vagal sensory signaling impairs HPC-dependent memory function in rats, and that the medial septum is an anatomical relay linking vagal signaling to the dorsal HPC [39]. The medial septum is the primary source of ACh input to the HPC [44], and WD consumption disrupts gastrointestinal vagal signaling [29,35,36,57]. Thus, our working hypothesis is that consumption of a WD, especially during early life periods of development, impairs vagus nerve signaling, which in turn gives rise to blunted ACh neurotransmission and associated deficits in HPC-dependent memory function (Figure 1). This hypothesis is strongly supported by our recent results [58], revealing that early life WD, septal ACh neuron ablation, and vagotomy lead to similar reductions in HPC cholinergic signaling and impaired memory function.

While findings from animal studies form the basis of this hypothesis, no clinical research to date has demonstrated that early life WD consumption leads to long-lasting impairments in memory function in humans. However, it is important to note that following a Western dietary pattern during adulthood (age 60+ years) is associated with smaller hippocampal volume in humans [59] and, furthermore, a growing body of evidence indicates the critical role of the vagus nerve in memory function in humans [37,40,60]. Future clinical studies are imperative for better understanding these potential connections, especially related to early life WD consumption and long-lasting effects on memory function.

### Dopamine

Dopamine (DA) is a neuromodulator involved in numerous behaviors, including motor control and positive reinforcement-based learning [61–63]. Midbrain dopaminergic neurons of the substantia nigra and ventral tegmental area (VTA) project broadly throughout the brain, including to the hypothalamus, cortex, HPC, and striatum [64,65]. Additionally, norepinephrine-producing neurons of the locus coeruleus (LC) can co-release DA [66] and innervate the HPC and striatum [67,68]. This section emphasizes how WD consumption mechanistically alters DA systems involved in reward and memory with a particular focus on projections to the striatum and HPC based on the susceptibility of these regions to WD-associated dysfunction, along with their critical roles in learning, memory, and reward-driven behavior.

VTA DA axons have been studied extensively in the context of their projections to, and regulation of, striatal spiny projection neurons. These striatal projection neurons can be divided into ‘indirect pathway’ projection neurons, with targets expressing DA type 2 receptors (D2Rs), and ‘direct pathway’ projection neurons, with direct target neurons expressing DA type 1 receptors [69]. Patterns of VTA neural activity communicate to these striatal projection neurons, acting in concert with excitatory cortical inputs in a spike timing-dependent manner to modulate striatal DA signaling, which in turn influences mesolimbic network dynamics to broadly influence cognitive processes [69,70]. DA release in the ventral subregion of the striatum, particularly the nucleus accumbens, is critically involved in reward-based learning and habit formation and has been closely studied in the context of substance abuse disorder [62,71,72]. An accumulating body of evidence links WD consumption and diet-induced obesity with disrupted signaling within this midbrain-striatal mesolimbic circuit that encodes reward-related learning [73–75].

LC-derived DA is implicated in learning and memory, particularly via LC projections to the HPC. DA is elevated in the HPC when rodents inspect novel objects [76], and photostimulation of DA synthesis-promoting tyrosine hydroxylase positive (TH+) CHR2 (channelrhodopsin-2) LC axons improved the performance of mice in a novel location recognition task, an effect abolished by hippocampal D1 receptor antagonism [77]. Another group found that optogenetic stimulation of LC TH+ axons in mice after training on a food location memory task improved the persistence of memory for that food location, and these performance enhancements were also D1 type receptor-dependent [78]. Five-month HFD exposure (60% kcal from fat) in mice induces inflammation and microglial immunoreactivity in the LC [79], and rats maintained on an HFD containing 45% kcal from fat for 30 weeks exhibit significant reduction of TH-expressing cells in the LC [80]. These findings implicate diet-induced effects on DA networks related not only to reward circuitry but also memory consolidation. While norepinephrine that is produced in the same LC neurons as DA also plays a role in cognition [81,82], thus far there is limited-to-no evidence indicating norepinephrine plays a role in vagally mediated WD-driven changes in memory function, affective processes, and reward-related behavior. Taken together with findings from our group and others that WD consumption disrupts hippocampal memory function [49,83], aberrant LC DA signaling may be playing a functional role in these cognitive deficits.

Palatable foods are potent reinforcers for learning, particularly commonly consumed WD foods that synergistically combine sugars and fats [84,85]. In humans, PET scans employing radiolabeled DA antagonists revealed that milkshake consumption elicits DA release in the HPC and nucleus accumbens during both orosensory and post-ingestive stages [86]. Consistent with these nutrient-driven responses, rodents with 6-hydroxydopamine-lesioned midbrain DA neurons do not eat—even to the point of ultimately starving to death—and repletion of DA rescues animals from this phenotype [87,88]. Further, the levels of DA release in the nucleus accumbens at the onset of food consumption is higher in fasted animals than in fed animals [89]. Additionally, LC TH+ neurons in mice have been shown to exhibit reduced activity during consumption, and the magnitude of this response is influenced by prandial state [90]. Thalamic leptin-receptor expressing neurons, which play a role in responding to metabolic cues and maintaining energy homeostasis, strongly innervate both the VTA and LC, implicating both DA systems in homeostatic networks that regulate energy balance [91]. These findings collectively highlight that the mesostriatal and potentially the LC DA systems potently respond to nutrients, that these responses are essential for motivation driving homeostatic food consumption, and that this system is modulated by energy balance status.

In addition to influencing homeostatic nutritive processes (those required to obtain the basic nutrition required for survival), DA signaling is strongly associated with hedonic food-motivated behaviors and the rewarding properties of palatable food [92]. When animals are exposed to unexpected food rewards, VTA neurons fire in bursts resulting in transient increases in DA release in the nucleus accumbens. However, after repeated and predictable exposure to a specific food reward, this response to the primary reward of food consumption attenuates, and the phasic DA response shifts to the cues that reliably predict the food reward, such as the smell of food and/or to otherwise neutral stimuli that are experimentally conditioned to precede palatable food access [62,93]. These DA responses are behaviorally meaningful, as food cues alone can promote food consumption in otherwise sated rats and in humans, indicating that reward prediction networks contribute to hedonic eating in the absence of hunger [94–96]. In addition to involvement in motivation to obtain and consume palatable foods, in humans, striatal DA release levels correlate with pleasantness ratings of meals [97], and in rodents, VTA DA activity and striatal DA binding scale in magnitude with food palatability [98].

Chronic exposure to WD and associated diet-induced obesity have each been strongly linked to perturbations in DA molecular machinery in the brain in humans and animal models alike. For example, early human PET imaging findings demonstrated inverse correlations between BMI and striatal D2R availability, as measured by radioactive competitive antagonist displacement [99,100]. However, other groups have since found results that conflict with these earlier characterizations of D2R availability and striatal DA tone, perhaps due to the varying radioligands and study design [101,102]. A recent meta-analysis of several D2R availability studies in humans concurs with the reduction in D2R availability results and posits reward insensitivity as a potential driver of obesity etiology [103]. Research in animal models has further characterized the direct link between WD exposure and D2R signaling, validating that prolonged WD exposure (e.g. 5+ weeks) reduces striatal D2R protein expression [104,105]. This WD-induced D2R downregulation may functionally blunt the sensitivity and cellular responses to reward-related DA release. D2Rs are also known to be expressed presynaptically on DA releasing neurons, and these ‘autoreceptors’ are implicated in regulating the DA release and activity of these neurons [106]. Impairments in receptor expression as a result of WD consumption may result in blunted DA signaling associated with rewards, potentially leading to overconsumption of palatable foods to compensate [107].

In addition to the D2R phenotype, WD and obesity are also associated with decreases in striatal expression of DA transporter, a molecule responsible for the reuptake of DA into presynaptic VTA DA neuron axon terminals [108–110]. Reduction in DA transporter expression is correlated with obesity in humans, as well as in rats given *ad libitum* access to a milk chocolate-based nutrition shake for 30 days [108,109]. Downregulation of DA transporter could further result in a deficit of vesicular DA repletion, and subsequent reductions in DA tone. Indeed, striatal tonic DA signaling as measured by radioactive raclopride displacement has been shown to be reduced in people with obesity and the postprandial striatal DA elevation seen in healthy individuals is absent with obesity [111,112]. These findings imply that—not only are tonic levels depleted—but phasic DA responses to food rewards are attenuated in obesity. WD exposure in rodents phenocopies these obesity-related DA depletions, as phasic DA release at consumption onset and amphetamine-induced DA release in the nucleus accumbens are blunted in rodents maintained for 12 weeks on a 40% kcal from fat HFD or a cafeteria diet [73,74]. Taken together, these findings suggest WD-induced obesity results in pronounced impairments of presynaptic DA release, reuptake, and postsynaptic DA sensitivity in both humans and experimental rodent models.

What are the cognitive and behavioral implications of altered dopaminergic transmission and cellular responsivity associated with WD consumption and/or obesity? A leading theory draws on connections between the obesity and addiction fields, positing that WD overconsumption can result in ‘food addiction’ [113]. While the validity of food addiction theory is still widely debated, there are undoubtedly mechanistic overlaps and parallels that warrant discussion. For example, addiction to drugs of abuse induces similar hypodopaminergic phenotypes as diet-induced obesity [113]. These deficits in DA release and sensitivity in the context of drug addiction have been classified as reward deficiency syndrome [114]. In recent years, reward deficiency syndrome’s applications have been expanded, such that it is now associated with a slew of cognitive outcomes including obesity, impulsivity, and binge-eating disorder [107]. Further, there are correlations between genetic markers for DA machinery, reduced striatal reward responses, BMI, and food addiction scores in humans, further underlying the overlap of these systems [115]. In rats, diets rich in fats or sugars reduce ventral striatal D2R expression and increase impulsive choices [116]. Further, WD-fed rats trained on a Brain Stimulation Reward paradigm, in which the rats self-initiated electrical stimulation of the lateral hypothalamus, needed a significant increase from their trained reward threshold following striatal D2R knockdown [105]. These results lend credence to the hypothesis that WD exposure induces changes in reward sensitivity through mechanistic disruption of DA.

### Vagus nerve hypothesis related to dopamine

What is responsible for diet-induced DA perturbations in the brain that lead to disruptions in cognition? Following off of work showing that HFD consumption disrupts vagal innervation of the caudal brainstem [30–32], there is growing evidence that vagal control of DA networks may contribute to the cognitive phenotypes associated with diet-induced obesity [36,117,118]. Although classically assumed to inhibit reward circuits and suppress motivated behavior based on stimulation of satiation and satiety, more recent evidence has indicated that vagus nerve signaling plays a role in stimulating reward circuits and promoting motivated behavior [117,118]. Distinct vagal sensory neuron populations detect sugars versus fats and may exert additive effects on reinforcement in the context of eating to promote overconsumption [36]. In fact, optogenetic stimulation of vagal sensory output via the nodose ganglia is sufficient to induce DA release from midbrain DA neurons in the substantia nigra [118]. Vagal stimulation has also been shown to affect LC firing patterns, with evidence linking VNS-stimulated LC activity in the context of epilepsy treatment [119,120].

With preliminary evidence that vagus nerve activity influences mesolimbic DA networks and the behavior of LC neurons, further work characterizing the way(s) chronic and acute WD consumption affect this network is needed. Based on findings that vagal projections in the brainstem are attenuated with WD [30–32], this may increase the threshold or magnitude of peripheral nutrient sensation required to induce mesolimbic DA release, which would be consistent with the findings of decreased reward sensitivity. As for the LC, changes to the firing patterns and DA release of these neurons due to diet may disrupt episodic memory formation and satiety networks, yet future studies are needed to directly evaluate this hypothesis (depicted in Figure 1).

### Serotonin

Serotonin (5-HT) is a neurotransmitter produced in the raphe nuclei of the brainstem with wide-ranging projections throughout the brain, implicating it in a broad array of physiological and behavioral functions including stress responses, learning and memory, and food intake [121,122]. While serotonin is produced centrally, it can also be synthesized peripherally, and dietary interactions with both the central and peripheral serotonergic system are common. For example, long-term WD consumption has been shown to impact the serotonergic system in various ways, affecting behaviors such as anxiety-like behavior, depression, and learning and memory in a wide variety of model systems [123–131].

A wealth of research suggests that chronic exposure to HFD can increase anxiety-like and depression-like behaviors [19,132–135], and a growing body of evidence suggests that the central serotonergic system is involved [123–125,136]. For example, Xia and colleagues [124] found that chronic consumption of a HFD (40% kcal from fat) increased anxiety-like and depressive behavior across a range of tests in mice. Male and female HFD-fed mice spent significantly less time in the center zone in the open field test, made significantly fewer entries into the open arm in the elevated plus maze, and showed a wide range of depression-like behaviors such as immobility in the forced swim test and tail suspension test [124]. These behavioral changes were associated with blunted activity of agouti-related peptide (AgRP) neurons in the hypothalamus, which project onto 5-HT3 receptor-expressing neurons in the bed nucleus of the stria terminalis (BNST). Infusion of a selective 5-HT3 receptor antagonist into the BNST rescued HFD-induced anxiety-like and depressive behaviors [124]. Similarly, others have shown that HFD-induced anxiety and depression in mice can be prevented with chronic administration of a selective 5-HT reuptake inhibitor [125].

Karth and colleagues [123] used a knock-in mouse line to reduce brain 5-HT levels via a mutation in the brain 5-HT synthesis enzyme tryptophan hydroxylase 2 (Tph2). Long-term HFD exposure (22 weeks; 39.7% kcal from fat) significantly increased anxiety-like behavior in the open field test in both wild-type and knock-in mice, with HFD-exposed animals making significantly fewer entries into the center zone. Additionally, knock-in mice fed a HFD had significantly more immobile episodes during the forced swim test when compared to wild-type mice fed a HFD [123]. However, when experiments were repeated with female mice no differences were found in the forced swim test, and anxiety-like behavior was only altered via reduced entries into the open arms during the elevated plus maze task, suggesting that sex may play an important role in mediating HFD-induced effects on serotonergic system-associated cognition [136].

Despite the evidence discussed above suggesting that reduced 5-HT levels are linked with—and possibly exacerbate—anxiety- and depression-like behavioral phenotypes due to WD consumption, it is important to recognize that overall results are mixed, as other research has found short- and long-term HFD exposure decreases anxiety-like behavior [126,137–139]. In adult male rats, 12 weeks of HFD consumption (60% kcal from fat) reduced anxiety-like behavior in the elevated plus maze task, marked by increased time spent in the open arm and increased exploratory behavior in the open field test [126]. Additionally, HFD-fed animals exhibited decreased latency to reach the platform in the Morris Water Maze both 2 h and 24 h after training, suggesting an improvement in learning acquisition and memory retention in these animals relative to controls [126]. These behavioral changes were accompanied by increased 5-HT metabolism in the HPC, suggesting long-term HFD exposure drives both behavioral changes and physiological changes in the serotonergic system [126]. However, other studies have found no changes in anxiety-like behavior in response to 8- or 12-week consumption of HFDs high in unsaturated fat (from olive oil) in male rats and mice, in contrast to increased anxiety-like behavior for groups consuming saturated fat-rich HFDs [140,141]. Another study reported a selective increase in anxiety-like behavior in male (but not female) rats as a result of short-term WD exposure (postnatal days 24-42) [142], indicating a heterogeneity in behavioral phenotypes in males. Furthermore, in female rats palatable food in the form of sucrose provided through a limited sucrose intake paradigm reduced indicators of anxiety- and stress-related behaviors in an estrous cycle-dependent manner [143], and WD consumption in male rats prevented the anxiogenic effects of early life short-term stress [144]. The underlying drivers of the varied anxiety-related responses to WD consumption are likely influenced by diet composition (e.g. saturated versus unsaturated fat content), sex-related differences, timing of diet exposure, and degree of exposure to environmental stressors.

In rhesus macaques, long-term access to a hypercaloric diet (HCD; 36% kcal from fat) led to deficits in reversal learning, with animals making more perseverative errors in a discrimination reversal learning task after 6-month HCD exposure when compared to pre-HCD exposure [127]. Interestingly, treatment with a 5-HT2C receptor agonist (WAY163909) significantly reduced perseverative errors, as well as increased the total number of correct responses [127]. However, when WAY163909 was co-administered with a 5-HT2C receptor antagonist, the rescue of perseverative errors was blocked [127]. Perseverative behavior has been shown to play a role in addiction, suggesting that serotonin could also be interacting with hypercaloric diets (and therefore WDs) to affect reward motivated behaviors.

Koopman and colleagues [128] found that hypercaloric snacking, especially on HFHS foods, can affect the serotonergic system in humans. Twenty-five healthy participants followed a 6-week hypercaloric diet, increasing HFHS food consumption via increased meal size or frequency, after which serotonin transporter binding was assessed via single photon emission computed tomography (SPECT). SPECT imaging revealed that serotonin transporter binding in the hypothalamus was significantly lowered when surplus calories were consumed between meals (increased meal frequency) when compared to surplus via increased meal size [128]. Additionally, overall serotonin transporter binding was decreased by 30% following 6-week consumption of an increased frequency, hypercaloric diet [128]. While this study did not test behavioral effects associated with HFHS food consumption and changes in the serotonergic system, it suggests that previous work in various model organisms may well translate to humans.

Finally, the effects of exposure to a WD on 5-HT signaling and associated behaviors can be especially pronounced during development. Ceasarine and colleagues [129] found that maternal HFD (45% kcal from fat) caused sex-specific behavioral outcomes in offspring, with female mice showing reduced sociability in a social preference test and male mice showing reduced preference for sucrose in a preference test. Male offspring exposed to maternal HFD also had significantly decreased fetal forebrain and placental 5-HT mRNA expression, while in female offspring 5-HT expression was unaffected [129]. These findings were echoed in human data, where Ceasarine and colleagues [129] found that fetal brain and placental 5-HT protein levels were negatively correlated with maternal decidual triglyceride accumulation (used as a proxy for maternal dietary fat consumption), an effect again only seen in males. Their research goes on to suggest that endotoxin accumulation and perinatal inflammation are implicated in these changes.

Serotonin signaling in nonhuman primates is similarly affected by maternal HFD exposure, with fetal offspring showing increased Tph2 (the rate limiting enzyme for 5-HT synthesis) and 5-HT1A receptor (the inhibitory 5-HT1A receptor) mRNA expression in the rostral dorsal raphe [130]. Female offspring (age 4 months) exposed to maternal WD (diet with 32% kcal from fat plus calorically dense treats) exhibited increased anxiety-like behavior when exposed to threatening novel objects, further suggesting an important role for sex [130]. Similar research has found that maternal WD (diet with 36.6% kcal from fat plus calorically dense treats) increases anxiety-like and stereotypic behaviors in both male and female 11-month old nonhuman primates, accompanied by reduced Tph2 mRNA expression in the dorsal raphe. Postweaning standard chow exposure was insufficient to rescue either behavior or Tph2 mRNA expression [131]. Taken together, current research suggests that exposure to WDs (particularly ones that are high in fat) is associated with increased anxiety-like and depressive behavior, changes which may be mediated via the central serotonergic system. Future research is needed to further elucidate the role that sex and developmental timing of HFD exposure may play in alterations to the serotonergic system.

### Vagus nerve hypothesis related to serotonin

Research has shown that vagus nerve activity plays a mediating role in the use of selective serotonin reuptake inhibitors (SSRIs) to treat depression [145,146]. For example, McVey Neufeld and colleagues [145] showed that treatment with an SSRI is sufficient to increase activity in VANs in the gut in mice. Importantly, a subdiaphragmatic vagotomy abolished the improvements in depressive-like behavior seen with oral administration of SSRIs as measured by the tail suspension test, suggesting that the behavioral effects of SSRIs depend on the function of the vagus nerve in mice [145]. Similarly, another study found that fluoxetine’s (an SSRI) effect on depressive behavior is mediated via the vagus nerve, and furthermore that it increases mRNA expression of 5-HT receptors (1A, 2B, and 3C) in the HPC [146]. VNS has been used as a treatment for depression, specifically for the treatment of resistant depression [147]. VNS has been shown to increase the firing rate of 5-HT neurons in the dorsal raphe [148,149], with Manta and colleagues [149] showing that this is accomplished indirectly via increased activation of noradrenergic neurons in the LC which project onto 5-HT dorsal raphe neurons. Furmaga and colleagues [150] have also shown that the serotonergic system is required for VNS-mediated effects on anxiety-like and depression-like behavior, with lesioning of central nervous system serotonin neurons abolishing the reduction of anxiety-like and depression-like behavior observed with repeated VNS. Building on these established links between vagus nerve activity, the serotonergic system, and mood-related behaviors, we posit that dietary-induced perturbations in vagus nerve function may adversely affect serotonin-mediated behavioral phenotypes (depicted in Figure 1).

Indeed, serotonin has also been shown to affect feeding behavior and obesity [29], but little is known about the direct role that WD exposure may play on the vagal influence over serotonergic systems. One study found that VNS in obese minipigs resulted in significant weight loss, a reduction in food intake, and an increase in resting energy expenditure [151]. Although cognitive outcomes were not measured in this study, these metabolic changes were accompanied by a significant increase in serotonin transporter binding potential in the midbrain [152]. Another study by Troy and colleagues [153] found that while HFD exposure (3 days–8 weeks; 60% kcal from fat) in rats did not directly affect the response of gastric VANs to 5-HT, it reduced the ability of glucose to amplify these effects, suggesting that glucose-dependent vagal afferent signaling is dampened with HFD exposure. Ultimately, while there is significant research connecting vagal signaling with serotonergic effects on cognitive outcomes, more research is necessary to untangle how exposure to an HFHS diet may interact with these systems to ultimately drive behavioral and cognitive changes.

## Conclusion

While the connections between food consumption, obesity, and cognition are complex and multifaceted, in this review we propose that consuming a high-fat & high-sugar WD has deleterious effects on metabolic and cognitive outcomes by modulating the function of, and signals carried by, the vagus nerve from the gut to the brain. Mechanistically, we hypothesize that these vagally initiated diet-induced changes can alter central acetylcholine, dopamine, and serotonin signaling. The timing (e.g. early life versus adulthood) and duration of WD consumption yield undiscovered heterogeneity in the severity and types of behavioral effects observed from various WD dietary models.

Here we have reviewed key alterations in central neuromodulators and vagal signaling, but a large and growing body of evidence indicates that WD consumption can also affect a multitude of other peripheral factors that influence the brain, including the gut microbiome, gut barrier integrity, peripheral inflammation, as well as the function and secretion of peripheral peptides and hormones (e.g. insulin, leptin) [30,49,154–160]. Such peripheral factors also contribute to diet-linked impacts on behavior and cognition—potentially by altering vagal signaling to the brain, and/or by acting through other means such as changing humoral signaling or BBB permeability. Future research that allows for comprehensive assessment of the cross-talk among multi-organ physiological systems is imperative to not only more concretely understand the purported role of the vagus nerve in WD-induced neurocognitive dysfunction, but also to unravel the relationships between peripheral and central factors more broadly, in diet-induced changes in physiology, behavior, and cognitive function.

Although we have focused our attention on the influence of WD consumption on central neuromodulators of cognitive function, it is also possible that peripherally produced neurotransmitters play a role in WD-induced changes in learning and memory, affective processes, and reward-related behaviors, especially given that over 50 gut bacterial strains thus far have demonstrated neurotransmitter-producing capabilities [161]. For example, Fujita and colleagues [162] found that the gut microbial taxon *Lactiplantibacillus plantarum* is capable of producing ACh in a gut microbiome model involving living Drosophila melanogaster larvae. Furthermore, while WD consumption increases levels of peripheral 5-HT produced from intestinal enterochromaffin cells [163], this WD-induced peripheral elevation has ultimately been tied with decreased 5-HT levels in the HPC and hypothalamus [164]. To our knowledge, there is a lack of research investigating how WD consumption could alter the functionality of peripheral neurotransmitters (including ACh, DA, and 5-HT), and furthermore how these changes are integrated to alter central neurotransmitters systems, and by extension, cognitive and behavioral processes.

We emphasize that a WD is heterogenous and complex in composition, both as used in animal studies and in real-life settings. In this review we have highlighted the general macronutrient composition of the diets used in the various studies discussed. However, some studies (including our own [49,83,165]) have incorporated WDs containing an assortment of food/drink options that the study subjects can consume freely as they choose, while others have used purified rodent diets with fixed macronutrient compositions without dietary choice. In addition to a literature lacking precise macronutrient composition information, knowledge about the amount and type of dietary fiber provided in the various types of diets is limited thus far. Given the growing body of evidence indicating that dietary fiber can play key roles on gut-brain axis signaling to alter behavioral and cognitive phenotypes [166–169], greater emphasis should be placed on these aspects of both experimental WDs and control diets in the future. These dietary differences may also contribute to the variability in WD-induced effects across studies. Future research distinguishing which aspect(s) of a WD drive more versus less deleterious effects is critical.

Collectively, through this review we have outlined a framework for conceptualizing how consumption of a WD can give rise to alterations in the cognitive domains of memory function, anxiety-like behavior, depressive-like behavior, and reward-associated behavior by changing the signaling profiles of critical brain-derived neuromodulators. Our central hypothesis is that impaired vagus nerve-to-brain signaling plays a key role in these WD-induced cognitive impairments. Recent advances in vagus nerve-targeted experimental approaches (e.g. more selective sensory lesioning, improved *in vivo* imaging applications), combined with enhanced *in vivo* detection of brain region-specific neurotransmitter signaling (e.g. novel fluorescent-based sensors) in rodent models offer a platform for future work to more systematically evaluate this hypothesis.

## Open Access

Open access for this article was enabled through an open access agreement between the Biochemical Society/Portland Press and the University of California.

## Abbreviations

ACh: Acetylcholine
AChE: Acetylcholinesterase
AgRP: Agouti-related peptide
α7nAChR: α7 nicotinic acetylcholine receptor
BBB: Blood-brain-barrier
BMI: Body mass index
BNST: Bed nucleus of the stria terminalis
CHR2: Channelrhodopsin-2
CCK: Cholecystokinin
DA: Dopamine
D2R: Dopamine type 2 receptor
HCD: Hypercaloric diet
HFD: High-fat diet
HFHS: High-fat, high-sugar
HPC: Hippocampus
LFD: Low-fat diet
LC: Locus coeruleus
NTS: Nucleus of the solitary tract
PET: Positron emission tomography
SPECT: Single photon emission computed tomography
SSRI: Selective serotonin reuptake inhibitor
TH: Tyrosine hydroxylase
Tph2: Tryptophan hydroxylase 2
VAChT: Vesicular acetylcholine transporter
VAN: Vagal afferent neuron
VNS: Vagus nerve stimulation
VTA: Ventral tegmental area
WD: Western diet
5-HT: 5-hydroxytryptamine or serotonin
